# Strengthening universal health: development of a nursing and midwifery education quality improvement toolkit

**DOI:** 10.1590/1518-8345.3229.3188

**Published:** 2019-12-05

**Authors:** Adelais Markaki, Jacqueline Moss, Allison Shorten, Cynthia Selleck, Lori Loan, Rhonda McLain, Rebecca Miltner, Patricia Patrician, Lisa Theus, Lilian Ferrer, Fernanda dos Santos Nogueira de Góes, Maria Teresa Valenzuela-Mujica, Rosa Zarate-Grajales, Silvia Helena De Bortoli Cassiani, Doreen Harper

**Affiliations:** 1 University of Alabama at Birmingham, School of Nursing, Birmingham, Alabama, USA.; 2 Pontificia Universidad Catolica de Chile, Escuela de Enfermería, Santiago, Chile.; 3 Universidade de São Paulo, Escola de Enfermagem de Ribeirão Preto, PAHO/WHO Collaborating Centre for Nursing Research Development, Ribeirão Preto, SP, Brazil.; 4 Universidad Autonoma de Mexico, Escuela Nacional de Enfermería y Obstetrícia, Mexico City, Mexico.; 5 Pan American Health Organization, Washington D.C., USA.

**Keywords:** Primary Health Care, Quality Improvement, Universal Health Insurance, Nursing Education, Nurse Midwives, Midwifery, Atenção Primária à Saúde, Melhoria de Qualidade, Cobertura Universal do Seguro de Saúde, Educação em Enfermagem, Enfermeiras Obstétricas, Tocologia, Atención Primaria de Salud, Mejoramiento de la Calidad, Cobertura Universal del Seguro de Salud, Educación en Enfermería, Enfermeras Matronas, Partería

## Abstract

**Objective:**

to present the development of a toolkit for education quality improvement in universal health and primary health care, targeting schools of nursing and midwifery in Latin American and Caribbean countries.

**Methods:**

an expert work group conducted a systematic literature review, selected key content and completed toolkit drafting, using an iterative consensus approach. International partners reviewed the toolkit. Cognitive debriefing data were analyzed, revisions and new tools were integrated, and the final version was approved.

**Results:**

twenty-two articles were identified and mapped as resources. The Model for Improvement, a data-driven approach to performance analysis, was selected for its widespread use and simplicity in carrying out the following steps: 1) organize a team, 2) assess improvement need regarding universal health and primary health care education, 3) set an aim/goal and identify priorities using a matrix, 4) establish metrics, 5) identify change, 6) carry out a series of Plan-Do-Study-Act learning cycles, and 7) sustain change.

**Conclusions:**

the Education Quality Improvement Toolkit, developed through stakeholder consensus, provides a systematic, and potentially culturally adaptable approach to improve student, faculty, and program areas associated with universal health coverage and access.

## Abstract (pt)

**Objetivo:**

apresentar o desenvolvimento de um kit de ferramentas para melhoria da qualidade da educação em saúde universal e na atenção primária à saúde em escolas de enfermagem e obstetrícia na América Latina e no Caribe.

**Métodos:**

um grupo de especialistas realizou uma revisão sistemática da literatura, selecionou conteúdos-chave e desenvolveu uma versão preliminar do kit, através de uma abordagem iterativa de consenso. Posteriormente, esta versão foi submetida a revisão por parceiros internacionais. Foram efetuadas análise dos dados obtidos em entrevista cognitiva, seguida da integração de revisões e de novas ferramentas até a se alcançar a aprovação da versão final.

**Resultados:**

vinte e dois estudos foram identificados e mapeados como recursos. O Modelo de Melhoria - uma abordagem baseada em dados para a análise de desempenho - foi selecionado devido a sua ampla difusão e simplicidade na execução das seguintes fases: 1) formação de uma equipe, 2) avaliação das melhorias necessárias na educação em saúde universal e atenção primária à saúde, 3) determinação de objetivos/metas e definição de prioridades com base numa matriz, 4) estabelecimento de métricas, 5) identificação de mudanças, 6) realização de uma série de ciclos de aprendizagem Planeje-Faça-Estude-Atue, e 7) manutenção das mudanças.

**Conclusões:**

o Kit de Ferramentas para Melhoria da Qualidade da Educação, desenvolvido a partir do consenso entre as partes envolvidas, representa uma abordagem sistemática, potencialmente adaptável às diversas culturas, para aprimorar aspectos do corpo discente, do corpo docente e de programas de educação relacionados com a cobertura e o acesso à saúde universal.

## Abstract (es)

**Objetivo:**

presentar el desarrollo de un kit de herramientas para la mejora de la calidad de la educación en salud universal y atención primaria de salud, dirigido a las escuelas de enfermería y obstetricia en los países de América Latina y el Caribe.

**Métodos:**

un grupo de trabajo de expertos realizó una revisión sistemática de la literatura, seleccionó el contenido clave y redactó las herramientas, utilizando un consenso iterativo. Socios internacionales revisaron el kit de herramientas. Se analizaron los datos de la entrevista cognitiva, se integraron las revisiones y las nuevas herramientas, y se aprobó la versión final.

**Resultados:**

veintidós artículos fueron identificados y registrados como recursos. Se seleccionó el Modelo para Mejoramiento, un enfoque basado en datos para el análisis del desempeño, por su uso generalizado y sencillez para realizar los siguientes pasos: 1) organizar un equipo, 2) evaluar la necesidad de mejora con respecto a la educación universal en salud y atención primaria de salud, 3) establecer el objetivo e identificar las prioridades utilizando una matriz, 4) establecer métricas, 5) identificar el cambio, 6) llevar a cabo una serie de ciclos de aprendizaje Planear-Hacer-Estudiar-Actuar, y 7) mantener el cambio.

**Conclusiones:**

el Kit de Herramientas para la Mejora de la Calidad de la Educación, desarrollado mediante el consenso de las partes interesadas, proporciona un enfoque sistemático y culturalmente adaptable en potencia para mejorar las áreas de los estudiantes, profesores y programas que se encuentran asociados con el acceso y cobertura universal de salud.

## Introduction

In 2016, the Pan American Health Organization (PAHO), in collaboration with three World Health Organization Collaborating Centers (WHOCCs) in Nursing and Midwifery, and the Latin American Association of Nursing Schools and Faculty (ALADEFE), conducted an *“Analysis of Nursing Education in the Region of the Americas towards Primary Health Care and Universal Health.”* Latin American and Caribbean (LAC) nursing and midwifery programs were surveyed regarding their preparation of graduates to promote Universal Health (UH), their orientation towards Primary Health Care (PHC), and focus on social determinants of health. Although various program strengths were identified, this collaborative work identified a need for ongoing monitoring, evaluation, reporting and development of quality improvement (QI) plans, to ensure graduate preparedness in PHC and UH. Recommendations included: 1) strengthening knowledge of integrating information technologies (IT) into healthcare, environmental and global health, emergency and disaster preparedness, complex and systemic thinking, problem solving and evidence-based care; 2) expanding use of clinical simulation and training experiences in PHC settings; and 3) adopting the principles of interprofessional education (IPE) using practical team-based experiences that reflect country specific healthcare contexts and priorities around PHC and UH^(^ 1 ^)^.

In the footsteps of this study, the PAHO/WHOCC in International Nursing at the University of Alabama at Birmingham (UAB) was tasked to develop *“Universal Health and Primary Health Care: a Plan for Nursing and Midwifery Education Quality Improvement.”* This plan included an education quality improvement (EQI) Toolkit to assist nursing and midwifery programs in evaluating and improving structure, process, and outcomes within the framework of transformative education and competency-based interprofessional collaborative practice (IPCP). The overarching goal for this international multi-centric partnership study was to improve UH and PHC in LAC countries. In this paper, we will describe the development, evaluation by partners, and revision of the EQI Toolkit aimed to guide QI activities in nursing and midwifery education.

### Transformative Education

In the era of a global economy, sweeping technological advances, growing disparities and increased focus on sustainability, higher education for health professionals is rapidly evolving. Pathways for transforming higher education to strengthen health systems in an interdependent and interprofessional world have been the focus of two simultaneous landmark reports^(^ 2 ^-^ 3 ^)^. Transformative education encompasses principles of critical thinking, team work, creative adaptation, integration of education into health systems, resource sharing, networking and partnerships^(^ 2 ^-^ 3 ^)^. Interprofessional education, where students learn from each other outside traditional disciplinary silos, is a pre-requisite to transformative education^(^ 3 ^)^. Preparing the future healthcare workforce for IPCP, different disciplines working effectively together in teams with patients, families, caregivers, and communities, is a prerequisite to delivering the highest quality care^(^ 3 ^)^. The High-Level Commission on Health Employment and Economic Growth, established by the United Nations (UN), WHO, and other agencies, recommends scaling up transformative and lifelong learning for the healthcare workforce^(^ 4 ^)^. To move forward the UN 2030 Sustainable Development Goals (SDGs) agenda, nurses and midwives must be prepared to practice and thrive in an ever-changing environment, assessing evidence and working collaboratively with other healthcare professionals to meet the needs of diverse populations.

Universal health (UH) access and coverage are two of the main indicators for ensuring healthy lives and promoting well-being for all (SDG #3). Defined as “the absence of geographical, economic, sociocultural, organizational, or gender barriers” universal access to health is achieved through “the progressive elimination of barriers that prevent all people from having equitable use of comprehensive health services determined at the national level”^(^ 5 ^)^. Universal health coverage (UHC) is a financial protection arrangement for health for all. It is often achieved through some form of national health insurance program to serve the needs of the population^(^ 6 ^)^. Closely tied to both concepts, PHC aims to achieve better health for all through reform in universal coverage, service delivery, public policy, and leadership^(^ 7 ^)^. Organizing health services around peoples’ needs and expectations, and increasing stakeholder participation are two key PHC mandates. Recently, the potential contribution of nurses and midwives towards achieving UH has been a focus of the Nursing Now Campaign^(^ 8 ^)^. Based on the premise that investment in nursing will enable nurses to achieve their full potential, nursing and midwifery education programs are urged to prepare graduates in promoting UH and PHC.

In order to prepare competent and skillful entry-level nurses and midwives in providing PHC and UH, educators must meet a minimum of core competencies. The *“Midwifery Educator Core Competencies”* ^(^ 9 ^)^ and the *“Nurse Educator Core Competencies”* ^(^ 10 ^)^ were developed in response to the World Health Assembly resolutions upon consultation with key partners. Both documents aim to support and guide educational institutions, worldwide, in modifying their competencies-based curricula within each country’s parameters, including diversity and availability of resources. Monitoring, assessment and evaluation of students and programs are listed as educator competencies for both nurses and midwives^(^ 9 ^-^ 10 ^)^ linking core competencies to the ongoing debate about improving educational outcomes in nursing and midwifery schools.

### Background - Nursing and Midwifery Education in Latin America and the Caribbean

Entry-level nursing and midwifery education in LAC countries reflects regional disparities in terms of geography, politics, economy, and culture^(^ 11 ^)^. Variation in levels of educational preparation affect workforce capacity, in both numbers and skill mix, which ultimately influences care quality. For example, some Eastern Caribbean countries have more than 40 professional nurses per 10,000 population, whereas most of the Spanish-speaking countries in the region have less than 10 per 10,000^(^ 12 ^)^.

Similarly, teaching and practicing midwifery varies greatly, with an ongoing debate over education and care models^(^ 13 ^)^. Midwifery can be practiced by obstetric nurses, nurse-midwives, professional midwives, and in some countries, by traditional midwives, with education ranging from lay midwifery to undergraduate and/or post-graduate preparation^(^ 14 ^)^. For countries with low numbers of skilled birth attendants and high maternal mortality rates, it has been recommended that Ministries of Health consider professional midwifery as a key to improving maternal and perinatal health^(^ 14 ^)^. Therefore, strategies to strengthen the quality of nursing and midwifery education are vital for establishing a competent workforce and must be set in the context of each country.

### Quality Improvement in Healthcare and Health Professional Education

Quality improvement, as we currently know it in healthcare and education, originated in 1939 with control charts to track variation in manufacturing defects and the “learning and improvement cycle” known in healthcare as the Plan-Do-Study-Act (PDSA) cycle^(^ 15 ^)^. Studying what is occurring and its variation patterns was viewed as a first step before intervening to improve quality. The impact of specific QI approaches varies, depending on the context in which they are implemented^(^ 16 ^)^. In general, success of a QI program is determined by an organization’s commitment to monitor, assess, improve, and embed continuous improvement into the organization’s culture for seeking higher levels of performance. Understanding systems and how they interact is critical, since improvement in one area of operations may induce unintended consequences, and even harm, in another area^(^ 17 ^)^. Focusing improvement efforts on customers who benefit from the system is fundamental, whether they be patients, students, providers, or teachers. A team approach, involving more than one discipline, builds creativity, enthusiasm, and commitment to the work. Focus on collecting and using data, constant monitoring, and tracking the same elements throughout the iterative PDSA cycles is paramount to successful QI endeavors. The WHO has defined QI as “An approach to improvement of service systems and processes through the routine use of health and programme data to meet patient and programme needs.”^(^ 7 ^)^ This versatile definition can be applied to both healthcare delivery and educational programs. Improving quality in healthcare requires an appreciation and understanding of system complexity, critical components, and most importantly, the cultural context and population served^(^ 18 ^)^. Educational program quality can be defined as the degree to which didactic and clinical education increases the likelihood of desirable outcomes, consistent with current knowledge. These educational outcomes should reflect competencies of nurses and midwives as they relate to providing patient care.

In light of the above priorities and needs for highly competent nursing and midwifery professionals who are well-equipped to promote UH and PHC, this article aims to present the development of an EQI Toolkit for schools of nursing and midwifery in LAC countries.

## Method

**Figure 1:**
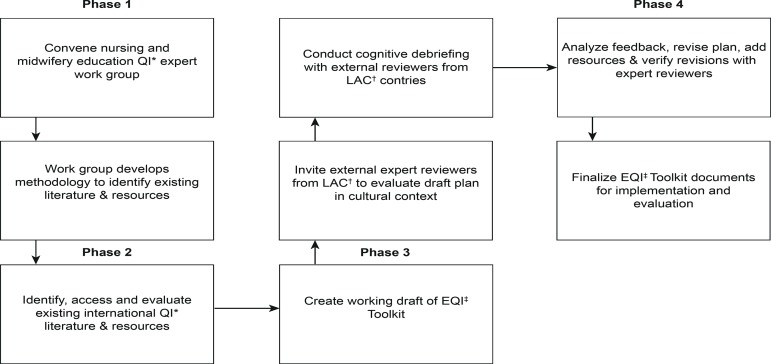
Development process of the EQI Toolkit *QI: Quality Improvement; †LAC: Latin America and Caribbean; ‡EQI: Education Quality Improvement

Following the Standards for Quality Improvement Reporting Excellence (SQUIRE), this multi-centric study is outlined in sequential phases (Figure 1).

### Phase 1 - QI expert work group

A group of 10 faculty with expertise in the areas of QI, improvement science, program evaluation, competency-based education, IPE, IPCP, midwifery, primary health care, rural care, and global health were assembled. This expert group was tasked with reviewing previous work on educational resources in the LAC region, as well as the broader literature, to develop a plan for self-directed QI among nursing and midwifery schools/programs. As a starting point, an extensive survey carried out across LAC nursing school programs regarding preparation of graduates to promote UH and PHC was reviewed for baseline data and recommendations^(^ 1 ^)^. For consistency with this previous PAHO commissioned work, the term UH was used as encompassing both universal access to health and universal health coverage. Donabedian’s model^(^ 19 ^)^ provided the framework for toolkit design and was operationalized through the QI process; a data-driven, formal approach to performance analysis in nursing and midwifery educational programs and the systematic efforts to improve it^(^ 16 ^)^.

### Phase 2 - Literature review

**Figure 2:**
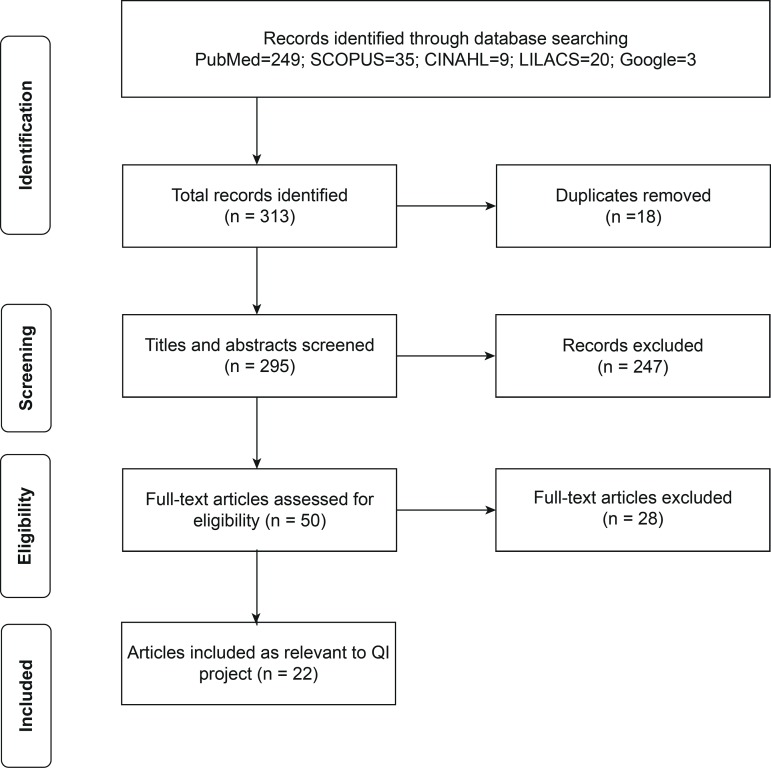
Literature search and inclusion criteria* *Source: Moher D, Liberati A, Tetzlaff J, Altman DG. The PRISMA Group (2009). Preferred Reporting Items for Systematic Reviews and Meta-Analyses: The PRISMA Statement. PLoS Med. 2009;6(7):e1000097. doi:10.1371/journal.pmed1000097

A systematic literature search on the topic of QI, as it relates to nursing and midwifery education or nursing and midwifery education programs, was carried out in five databases; PubMed, Scopus, the Cumulative Index to Nursing and Allied Health Literature (CINAHL), the Latin American & Caribbean Health Sciences Literature (LILACS), and Google/PAHO. Search terms included: “education, nursing, midwifery, baccalaureate, program accreditation, quality improvement, standards, Latin America, Caribbean”. A total of 313 peer-reviewed full-text articles, published within the last 10 years (from 2010 to 2018) in English language were retrieved. Search strategy results are presented in the PRISMA flow chart (Figure 2).

Two investigators from the phase 1 expert group conducted independent title/abstract/full text reviews, discussed disagreements and a consensus was met. A total of 50 articles were screened for the following inclusion criteria: 1) focus on an educational program or school of nursing or midwifery; 2) focus on monitoring or evaluation or assessment of quality or implementation of a QI project; and 3) focus on a model or conceptual framework in relation to accreditation, competency-based education, IPE, IPCP or transformational education. Exclusion criteria were: 1) focus on a healthcare setting; 2) clinical practice focus; and 3) no full-text available online.

### Phase 3 - EQI Toolkit development

With surveyed areas for improvement in mind, the same 10-member expert group: 1) identified existing QI resources for nursing and midwifery educational programs, and 2) prepared the model for a QI plan that could be tailored by LAC schools to address specific weaknesses in UH and PHC. Development of this model evolved into the EQI Toolkit with a target population of all nursing and midwifery schools offering undergraduate programs in the LAC region. Because of substantial educational differences among programs, it was clear that the toolkit would need to be adaptable to country/language/culture/setting, plus be user friendly and simple to use. For clarity, a glossary of terms with appropriate references was developed as an appendix to the EQI Toolkit.

The first toolkit version was developed using brainstorming and modified affinity grouping techniques. This process allowed the expert work group to generate, categorize, and choose among ideas by posting common themes on a flip chart^(^ 20 ^)^. For this project, ideas were recorded using a laptop and were immediately projected onto a screen for everyone to see and compare notes. Each work group member took responsibility for drafting content and tools for inclusion. An iterative process was used to select and connect the key elements to form a coherent resource for the next phase of review.

### Phase 4 - EQI Toolkit user evaluation and revision

To obtain user evaluation, the qualitative method of iterative processing cognitive debriefing was applied. Four international stakeholders were invited as reviewers through three partnering PAHO/WHOCCs. Individuals represented Brazil, Chile, and Mexico and were chosen for their diverse perspectives and technical expertise. They were asked to review the first version and to provide detailed comments, suggestions, and track-changes in the actual file. Further, reviewers were asked to complete the Cognitive Debriefing Form (Figure 3) during a live virtual meeting.

**Figure 3:** Cognitive Debriefing form

**Cognitive Debriefing Form**				
**Responding WHOCC:_________________________________________** **Document: *"Universal Health and Primary Health Care: A Toolkit for Quality Improvement in Nursing and Midwifery Education"*** Please answer the following questions in as much detail as possible. 1. Tell us about your impression of this tookit? 2. What are the top two strengths and weaknesses of this toolkit? 3. In general, is the structure clear, easy to understand and follow? 4. In general, is the content clear, easy to understand and follow? 5. Overall, is the toolkit relevant to your school's situation? 6. How likely are you to use this toolkit in your school?				
**Document Sections**	**Did you have difficulty understanding this section? (No/Yes -explain)**	**Would you revise this section? If yes, how?**	**Is this section relevant to your school's situation in regards to this topic?**	**Other comments?**
Background				
Introduction to Universal Health & Primary Health Care				
Introduction to Quality Improvement in Nursing and Midwifery Education				
Methodology				
Figure 1. Model for Improvement (MFI)				
Using the Model for Improvement (Steps 1-7)				
Examples of educational improvement for UH and PHC				
Appendix A (Self-Assessment Tool)				
Appendix B (Aim Statement)				
Appendix C (Prioritization Matrix)				
Appendix D-1 (PDSA Example)				
Appendix E (Case Study)				
Appendix G (Definitions)				

Two investigators asked questions and recorded responses. For two of the Spanish speaking respondents, questions and answers were translated back and forth by the Spanish speaking investigator. Respondent behaviors, such as asking for clarifications, being ambivalent or skipping items were recorded. Any difficulties experienced by subjects were used to modify the first toolkit version, thus making data collection an iterative process. Qualitative content analysis of cognitive debriefing data from the international reviewers was applied. In addition, the reviewers’ electronic track changes and added comments were also considered as part of the review process. Last, reaching consensus was used when reconciling reviewers’ input and data in order to agree on a final version of the EQI Toolkit.

### Ethics

The UAB Institutional Review Board (IRB) designated this project as Not Human Subjects Research (IRB-300002006). The international review process was overseen by the leading investigator who safeguarded ethical standards of conduct and that all review comments were carefully considered.

## Results

### Literature review

A total of 22 articles met all inclusion criteria and are listed in Figure 4 as existing resources for this project, along with key relevant points. Type of study and level of evidence was appraised based on the hierarchical classification by Melnyk and Fineout-Overholt^(^ 21 ^)^.

**Figure 4:** Literature review articles according to type of study, level of evidence, implications and resource theme

Author (Year)	Country/ Setting/ Population	Type of study/Level of evidence*	Key findings / Implications	Resource theme
Andresen K & Levin P (2014)^(^ 22 ^)^	USA/ BSN^†^ nursing program clinical experience	Descriptive study / Level VI Curriculum improvement project	• Augmenting evaluation tools and processes for clinical learning required faculty collaboration • Collaborative partnerships yielded valuable lessons • Project outcomes include expanded clinical capacity, increased variety of clinical learning experiences, and improved quality of clinical experiences	Curriculum plan development
Armstrong GE et al. (2009)^(^ 23 ^)^	USA/ Pilot nursing school in phase 2 of ^‡^QSEN project	Descriptive study / Level VI Curricular redesign using the ^§^COPA model	• Shared values between competency-based curricular models and ^‡^QSEN initiative • ^‡^QSEN competencies enhance graduates' skills in ^‖^QI and safety • Synergistic framework of ^§^COPA practice and ^‡^QSEN content competencies	Curriculum plan development
Brown JF & Marshall B (2008)^(^ 24 ^)^	USA/ Nursing department in a historically black university	Descriptive study / Level VI ^¶^QEP using the fishbone diagram as framework	• Effectiveness of **CQI approach in increasing ^††^NCLEX-RN pass rates, improving student advisement, and increasing student satisfaction • Process of ^¶^QEP is recursive rather than linear, involves review of best practices	**CQI - Accreditation
Cassiani et al. (2017)^(^ 1 ^)^	25 Latin American & Caribbean countries/ Schools of nursing	Descriptive study / Level VI International cross-sectional survey	• Heterogeneity in nursing education reflects disparities • Similarities represent opportunities for sustained advancement towards ^‡‡^UH • Nursing curricula generally include principles and values of ^‡‡^UH, primary health care, and transformative education (i.e. critical and complex thinking, problem-solving, evidence-based clinical decision-making, lifelong learning) • A paradigm shift in health sciences education is needed in response to population needs • Improvement areas for nursing graduates to be fully prepared for ^‡‡^UH	Assessment - Evaluation
Coffman S et al.(2015)^(^ 25 ^)^	USA/ ^†^BSN nursing program simulation learning	Descriptive study / Level VI	• In the concierge model of simulation, a core team of trained experts implement high-fidelity simulation scenarios followed by debriefing • Kirkpatrick model: evaluation of student satisfaction and learning	Implementation - Case Study
Ellis P & Halstead J (2012)^(^ 26 ^)^	USA/ Nursing education baccalaureate and graduate degree programs	Expert opinion / Level VII	• Accreditation is an ongoing process of **CQI • The Continuous Improvement Progress Report addresses all standards and key elements for Accreditation of Baccalaureate and Graduate Degree Nursing Programs	**CQI - Accreditation
Escallier L & Fullerton J (2012)^(^ 27 ^)^	USA/ School of nursing evaluation protocol	Descriptive study / Level VI	• Step-by-step process to develop an evaluation protocol with linkages to external criteria for evaluation of the plan itself • Analysis and feedback is essential to the ^‖^QI process	Assessment - Evaluation
Fater KH (2013)^(^ 28 ^)^	USA/ Undergraduate nursing school	Descriptive study / Level VI Gap analysis	• Gap analysis as a method to assess core competency in the curriculum • Labor intensive nature of data mining required • Competency-guided curricula could decrease content saturation and promote active learning	Assessment - Evaluation
Gonzalez-Chorda VM & Macia-Soler ML (2015)^(^ 29 ^)^	Spain/ Undergraduate nursing program - 2^nd^ year students	Descriptive study / Level VI	• ^‖^QI of the teaching-learning process through analysis of tools that evaluate acquisition of skills. • Learning activities that did not reach quality indicators: problem based learning, use of electronic registration system as a learning and assessment tool, collaboration between professors and students during clinical practice	Assessment - Evaluation
Halstead J (2017)^(^ 30 ^)^	USA/ Nursing education program	Expert opinion/ Level VII	• **CQI is a hallmark of the accreditation process • Identifies areas for improvement and plans to address those areas	**CQI - Accreditation
Hooper J & Ayars V (2017)^(^ 31 ^)^	USA/ 27 vocational and 61 professional nursing education programs in Texas	Descriptive study / Level VI Self-study report	• Interventions for improvement: revising admission and re-admission criteria, identifying at-risk students and providing remediation, revising and enforcing policies, updating curriculum, basing decisions on program evaluation data, evaluating use of standardized exams • Most effective interventions across all programs: identifying at-risk students earlier, providing timely remediation, and enforcing program policies	**CQI - Accreditation
James B et al. (2016)^(^ 32 ^)^	UK/ 3 nursing program campuses in Scotland with final year students on clinical placement	Qualitative study / Level VI ^‖^QI project (practicum)	• Time needed to acclimatise, socialise and conduct the Practicum • Timing of the Practicum within the curriculum • Student fears due to nature of assignment, attempt to change practice, and adjustment to unique type of assignment • Transformation; sense of achievement, acknowledgement of key improvement skills	Implementation - Case Study
Kaplan B et al.(2011)^(^ 33 ^)^	USA/ Simulation center of a university affiliated nursing school in Southeast 4^th^ year ^†^BSN students (team members) and Emergency Nurse Practitioner students (team leaders)	Descriptive study / Level VI Interdisciplinary simulation experience - pediatric mock code	• Use of patient simulators is an effective strategy for deliberate practice of skills and standardized exposure to limited scenarios • Debriefing reinforced the evidence and reviewed ^‖^QI and safety through error identification and patient consequences • Highly rated simulation for realism, concept clarification, increasing knowledge base, ability to function in the clinical setting, and increasing confidence in caring for a critically ill infant	Implementation - Case Study
Karagory, PM (2014)^(^ 34 ^)^	USA/ Large Midwestern university undergraduate nursing program/ sophomore level students	Descriptive study / Level VI ^‖^QI project on a skilled nursing unit at a long-term care facility	• Systems and ^‖^QI thinking can be successfully integrated early in an undergraduate curriculum, as evidenced by students' readiness to learn, self-reflection, and ability to embrace concepts, processes, and outcomes • Teamwork and leadership skill development • Clinical partners benefit and get motivated	Implementation - Case Study
McComb, SA & Kirkpatrick JM (2017)^(^ 35 ^)^	USA/ ^†^BSN program	Descriptive study / Level VI Embedding systems thinking and ^‖^QI content across a 4-year curriculum	• Exposes students to real world experiences that highlight relevance and significance of these skills in the health care context • This approach bridges the education-practice gap • Sustainability requires energized players and champions	Implementation - Case Study
Nugent, E & LaRocco S (2014)^(^ 36 ^)^	USA/ Accelerated second-degree baccalaureate entry program in a small liberal arts college	Descriptive study / Level VI **CQI; comprehensive review of program, faculty and graduates	• Several programmatic and curricular recommendations • Program review provided metrics/information on improvement areas • Listening to graduates provided insightful direction for clinical and curriculum changes	**CQI - Accreditation
Posey, L. & Pinzt C (2017)^(^ 37 ^)^	USA/ Second-degree ^†^BSN program	Descriptive study / Level VI Teaching and Transforming through Technology project	• Blended learning can: support teaching goals and address course challenges; provide independent learning activities outside the traditional classroom; increase opportunities for active learning; and improve digital literacy and lifelong learning skills • Development of a high-quality blended program requires an established quality framework to guide course design and evaluation.	Implementation - Case Study
Santos, M. (2012)^(^ 38 ^)^	Brazil/ Nursing education	Expert opinion / Level VII Overview of nursing education transformation within the US health system	• The ^§§^IOM report on Nursing Education for the 21st century emphasizes: Complexity of patient needs and care environment • Competencies to deliver high-quality care; leadership, health policy, system improvement, research, evidence-based practice, teamwork, collaboration • Competency in community/public health, geriatrics • Knowledge of technological tools and management of information systems	**CQI - Accreditation
Seibert, SA (2014)^(^ 39 ^)^	USA/ ^†^BSN management course/ Senior students	Descriptive study / Level VI Development of practice-based learning activities based on ^‡^QSEN competencies	• Assignments focus on systems level thinking and process evaluation of facility characteristics, team communication, unit based improvement • Reflective components evaluate comfort level with being an agent of change, unit climate for change, and confidence in delegation/ communication skills	Curriculum plan development
Sherrod, RA (2008)^(^ 40 ^)^	USA/ ^†^BSN program/ students in senior leadership course	Descriptive study / Level VI **CQI project in a rural, nurse-managed clinic	• **CQI can be applied to audit/revise reimbursement processes • Successful **CQI can support financial viability of an organization • Teaching students the value of reimbursement and documentation early on	**CQI - Accreditation
Wassef, ME (2012)^(^ 41 ^)^	USA/ Graduate nursing program/ 10 students enrolled in nurse educator specialty	Descriptive study / Level VI Implementing a competency-based electronic portfolio	• An interdisciplinary team used the Plan, Do, Study, Act QI model to • Develop a professional e-portfolio template • Use of electronic portfolios demonstrates student accomplishments, documents program and course outcomes	Implementation - Case study
Wolf ZR (2011)^(^ 42 ^)^	USA/ Urban, private university/ undergraduate and graduate nursing students	Descriptive study / Level VI Analysis and taxonomy of student complaints by type, outcomes, complainants.	• Undergraduate nursing students made the most complaints; failure and dismissal from program was the most frequent taxon, with grading, teaching, and testing the next highest • **CQI: 1) reduce number of future complaints by early intervention and process improvement activities, 2) meet national accrediting organization requirements.	**CQI - Accreditation

* Levels of evidence based on Melnyk & Fineout-Overholt (2011) [21]: Level I = systematic review or meta-analysis; Level II = randomized control trial; Level III = controlled trial without randomization; Level IV = case-control or cohort study; Level V = systematic review of qualitative or descriptive studies; Level VI = qualitative or descriptive studies; Level VII = expert opinion or consensus.

† BSN = Bachelor of Science in Nursing;

‡ QSEN = Quality and Safety Education for Nurses;

§ COPA = Competency Outcome and Performance Assessment;

‖ QI = Quality Improvement;

¶ QEP = Quality Enhancement Plan;

** CQI = Continuous Quality Improvement;

†† NCLEX-RN = National Council Licensure Examination for Registered Nurses;

‡‡ UH = Universal Health;

§§ IOM = Institute of Medicine

Content analysis of selected articles revealed the following themes: 1) assessment - evaluation; 2) continuous quality improvement (CQI) - accreditation; 3) curriculum plan development; and 4) implementation - case study. Key points relevant to QI efforts in schools of nursing and midwifery, with a focus on UH and PHC, are presented below, grouped according to above themes.

A*ssessment and evaluation* are essential to the QI process, reflected in select articles^(^ 1 ^,^ 27 ^-^ 29 ^)^. Despite the labor intensive nature of data mining, it is argued that gap analysis and feedback, as a method to assess core competency in the curriculum, could decrease content saturation and promote active learning^(^ 28 ^)^. Program evaluation is achieved through course reviews, student surveys, pre-and-post-program assessment of students’ knowledge/skills, and faculty interviews about their experiences with new teaching methods^(^ 27 ^,^ 29 ^)^. It is recommended that evaluation protocols include linkages to external criteria for evaluating the plan itself, while the teaching-learning process should be subject to ongoing QI^(^ 27 ^)^. This is consistent with findings from an extensive survey of nursing schools in LAC countries showing nursing program evaluation, student evaluation, and outcomes as the top priorities for QI initiatives^(^ 1 ^)^. Using an original self-assessment tool, the same survey revealed the heterogeneity of education, with a clear need to strengthen training in UH, PHC, and transformational education. Schools seeking accreditation are highly encouraged to develop periodic evaluations of curricula and programs with participation from their students, as well as to share the results with educational authorities and professional organizations^(^ 1 ^)^.

The *CQI-accreditation approach* is described in select articles^(^ 24 ^,^ 26 ^,^ 30 ^-^ 31 ^,^ 36 ^,^ 38 ^,^ 40 ^,^ 42 ^)^. It provides metrics and information to administrators and faculty regarding the rigor of programs and the potential of graduates. CQI as a hallmark of the accreditation process, identifies areas for improvement and allows for planning^(^ 30 ^)^. Thus, the Standards for Accreditation of Baccalaureate and Graduate Degree Nursing Programs in USA, required by the Commission on Collegiate Nursing Education (CCNE), are also examined as an ongoing CQI process^(^ 26 ^,^ 42 ^)^. Brown and Marshall^(^ 24 ^)^ use a quality enhancement plan (QEP) to assess key factors affecting program outcomes. They demonstrate CQI effectiveness in increasing NCLEX-RN pass rates, improving student advisement, and raising student satisfaction. Other effective interventions across programs include: early identification of students at risk, timely remediation, and program policy enforcement^(^ 31 ^)^. Of equal importance is surveying past graduates of their opinion and feedback about strengths and weaknesses of the school/program^(^ 36 ^)^.

For *curriculum plan development*, faculty collaborations and partnerships were instrumental in augmenting evaluation tools and processes for clinical learning, expanding clinical capacity, and improving clinical experiences^(^ 22 ^)^. Armstrong et al.^(^ 23 ^)^ use the Quality and Safety Education for Nurses (QSEN) competencies to enhance a competency outcome performance assessment (COPA)-based curriculum. Reflective components of practice-based learning activities based on QSEN competencies engage students in evaluating: 1) level of comfort with being a change agent, 2) unit climate for change, and 3) delegation and communication skills^(^ 39 ^)^.

*Implementation case studies* have shown that students’ readiness to learn and ability to embrace concepts, processes, and outcomes measurement is enhanced by early teaching of systems and QI thinking within an undergraduate curriculum^(^ 34 ^-^ 35 ^)^. Exposing students to real world experiences that underline the significance of these skills helps bridge the education-practice gap^(^ 35 ^)^. A study with United Kingdom student nurses showed that they can successfully overcome their fears and meet the QI practicum challenge, if adequate support mechanisms are in place^(^ 32 ^)^. Doing a practicum as a compulsory assignment in a large-scale cohort is achievable, if there is balance between motivation to learn and fear^(^ 32 ^)^. Students develop teamwork and leadership skills, while clinical partners are motivated by the students^(^ 34 ^)^. Adopting a blended learning approach, where face-to-face classroom and online teaching is combined with clinical experiences, along with incorporating IT through the use of electronic portfolios to demonstrate student accomplishments and document program and course outcomes, have shown promising results^(^ 37 ^,^ 41 ^)^. Furthermore, use of patient simulators and the concierge model of simulation can be an effective strategy for deliberate practice of skills and standardized exposure to limited scenarios^(^ 25 ^,^ 33 ^)^. Clinical simulation and debriefing are effective methods to incorporate the competencies as set forth by the Institute of Medicine (IOM) and the American Association of Colleges of Nursing (AACN); patient-centered care, interprofessional teams, evidence-based practice, clinical reasoning, patient safety, and practice across the life span^(^ 33 ^)^ . Subsequently, the EQI Toolkit has attempted to incorporate all of the above competencies.

### User assessment and regional variation

As part of the evaluation process, all four international reviewers answered questions using the Cognitive Debriefing Form (Figure 3) addressing strengths/weaknesses, clarity, ease of understanding, and relevance of the toolkit to their specific school/program. The overall impression was that the toolkit was easy to understand, user-friendly and relevant. Identified strengths were: use of a defined model for QI, the inclusion of structure, process and outcome as evaluation criteria, clearly stated definitions, and material that was relevant and up-to-date. Reviewers recommended expanding description of the PDSA cycle and strengthening linkages between the QI model and midwifery education. All reviewers suggested making the toolkit more culturally specific to their region, and tailoring or refinement of case studies. Minor modifications to wording and additional examples were offered to reduce ambiguity.

### Components of final EQI Toolkit

**Figure 5:**
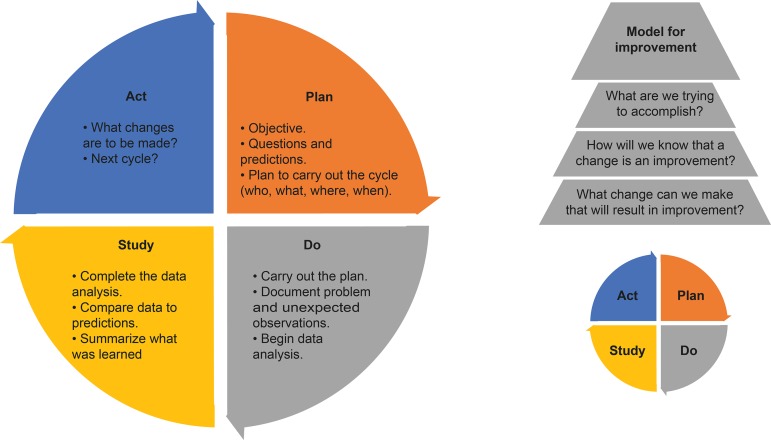
Model for Improvement and PDSA Cycle with steps Used with permission from Moen R, and Norman CL. Source: Langley GL, Moen R, Nolan KM, Nolan TW, Norman CL, Provost LP. The Improvement Guide: A Practical Approach to Enhancing Organizational Performance. 2.ed. San Francisco: Jossey-Bass Publishers; 2009. p. 24.

The toolkit includes an introduction to and definitions of UH, PHC, and IPCP concepts based upon WHO^(^ 6 ^)^ and PAHO^(^ 5 ^)^ documents. An overview of nursing and midwifery education presents the core competencies for educators^(^ 9 ^-^ 10 ^)^, followed by an introduction of QI in nursing and midwifery education. The expert group adopted the Model for Improvement (MFI) as the operational model for educational QI due to its widespread use and relative simplicity for end users. The MFI is described as a systematic method to effectively identify weaknesses or gaps in educational structures, processes, and outcomes. Starting with organizing a team to tackle the improvements and assess the current state, the MFI progresses to a series of questions that lead to an aim statement of what is to be accomplished. Measures are presented along with a strategy to determine whether an improvement has been made, identification of changes that may be tested to accomplish improvements, and iterative PDSA cycles to evaluate changes. The last step is how to sustain the change(s) and perhaps scale it to other programs or schools. Both the MFI and the PDSA cycles are depicted in Figure 5.

To accomplish the MFI and PDSA steps, six tools were incorporated as appendices to the toolkit. First, an instrument was developed for schools of nursing and midwifery to assess program components representing areas of excellence, as well as opportunities for improvement, specific to UH and PHC. The *“Universal Health and Primary Health Care in Nursing & Midwifery Education: A Self-Assessment QI Tool”* is envisioned for use in conjunction with other available tools to assess the current state. Using ‘yes’ or ‘no’ responses to statements, Part one of the tool assesses items related to the school’s structure, including mission, objectives and philosophy, as well as resources, infrastructure, external relationships and policies. Part two covers 14 items evaluating students’ and graduates’ professional competencies addressed in the curriculum, followed by 8 items related to curriculum model, teaching and learning strategies. Part three includes 6 items to evaluate the school, program and faculty outcomes, followed by guidance for tallying total scores.

Second, a *“Prioritization Matrix”* along with a completed example were included. Together, these items assist the improvement team to prioritize up to three items/areas requiring improvement, stemming from the results of the previously described self-assessment tool. The matrix guides the team in using weighed criteria to score improvement project options and use criteria-based evidence when choosing those to begin first. Priority criteria can be tailored for each school/program; cost, expertise, space, location, and organizational culture are some of the most frequently encountered.

Third, a *“PDSA Cycle Worksheet”* consisting of four sections along with a completed example was provided. The first section, *“Plan”,* asks the improvement team to detail the plan for a test of change; the improvement to be implemented, who will be involved, what are the components of the change, when will the change begin and end, where the project will occur, and predictions regarding the change. The team is asked to define all measures (process, outcome, balancing and system measures), name their data source and frequency of measure, and assign responsibilities. The second section, *“Do”,* reminds the team to determine the number of participants, carry out the change or test and collect planned data, while documenting problems and unexpected observations. In the third section, *“Study”,* improvement team members are guided to analyze data and summarize lessons learned, then compare data to a priori predictions and reflect on what was learned. Last, in the *“Act”* section, the team is prompted to refine the change based on what was learned, and consider whether to adapt, adopt, or abandon the change.

The fourth appendix includes two improvement exemplars for teaching UH and PHC competencies. The first one is a completed MFI/PDSA project illustrating an interprofessional team-based immersive simulation. It exemplifies language for problem statement, aim, measures, test of change (delivery of an interprofessional team), detailed project plan, and report of findings from the PDSA cycles. The second example offers an improvement project ready to be adapted and tested. Learning objectives, scenarios, questions to stimulate discussion, and prompts helping end users adapt the project to their courses are included.

The last two appendices of the EQI Toolkit represent the group’s collective knowledge, experience, and multicultural awareness. An extensive list of freely available web-based QI resources for nursing and midwifery educators and administrators was compiled. Special emphasis was given to resources from the LAC region by searching through the LILACS database, a comprehensive index of scientific and technical literature from Latin America and the Caribbean. Also, a glossary of terms with definitions of main concepts covered in the EQI Toolkit and relevant references was included to enhance understanding and clarity.

International reviewers were positive about using the QI process to evaluate their nursing programs, particularly if they didn’t currently use a standardized data collection and evaluation method. All reviewers suggested changes to make the toolkit more specific to the educational structure and culture of their region. Content was added to address the variation in nursing, nurse midwifery, and midwifery programs across LAC countries. Wording was changed to reflect cultural nuances and a PHC relevant case study was translated, culturally adapted, and applied to the PDSA model, addressing IPCP and nursing/midwifery competencies. During the last phase, a reconciliation list of QI resources was developed for final review and input with effort on including LAC specific resources in Spanish and/or Portuguese. Overall, feedback strengthened the usability of the toolkit.

## Discussion

The final EQI Toolkit is freely available through the UAB School of Nursing website^(^ 43 ^)^. Ultimately, the toolkit’s endorsement will be a measure of its comprehensiveness, relevance, and adaptability to different settings, institutions, and countries. Extension of its use to other types of programs, such as graduate, post-graduate, and interdisciplinary could signal greater adaptability and inclusiveness. Its potential for better educational outcomes and systematic QI is highly anticipated. Given that currently there is no mandatory accreditation for most nursing and midwifery programs in LAC countries, the EQI Toolkit could serve as a guide for institutions towards developing standards and procedures for ongoing, systematic internal quality control and improvement. Key higher education stakeholders can play an important role by providing strong incentives and building capacity at multiple levels (e.g., among individual faculty, interprofessional teams, across schools and institutions). Examples include QI education and training, technical assistance, ongoing mentoring or coaching, and financial support towards QI infrastructure development and staff. Improvement science coursework could also be introduced to undergraduate students.

Learning the science and application of improvement is becoming more pertinent to ​nurses and midwives around the globe. The Nursing Now^(^ 8 ^)^ global campaign has recently teamed up with the Institute for Healthcare Improvement (IHI) Open School to offer several free online modules on QI, program management, and culture change^(^ 43 ^-^ 44 ^)^. Although mostly targeting the healthcare environment, introductory and intermediate modules aim to build fundamental QI knowledge and skills in becoming a change agent.​ According to campaign leaders, nurses and midwives are well positioned to make innovative changes that not only improve their work practice environment, but also shift the universal health paradigm. After piloting and further refinement, LAC schools of nursing and midwifery could use this toolkit to improve capacity for preparing future professionals, according to their country and national health system needs. Long-term, the toolkit could perhaps, guide strategic planning initiatives and future directives for undergraduate programs and schools.

The EQI Toolkit was developed using a systematic and iterative process. Upon identifying relevant evidence-based literature from the LAC region, online resources stemming from professional associations, organizations and institutes were compiled into an accompanying appendix. Completeness and accuracy of data were assessed through: 1) reaching consensus after several iterations, 2) choosing expert group members and reviewers based on diverse perspectives and technical expertise, and 3) indirectly asking reviewers to endorse the content and providing them the final draft before its release. Cognitive debriefing revealed some strong positive comments in support of the developed plan for QI. Nevertheless, certain limitations exist. Systematic literature review was limited to English language articles with full-text availability, published within the last 10 years. All included articles were appraised at a low level of evidence indicating a deficit in available rigorous evidence. Pilot testing of the EQI Toolkit was not within this project’s scope and is planned for the future. Translation and linguistic adaptation into Spanish and Portuguese would be required to broaden the toolkit’s reach, once the original English version has been sufficiently pilot tested and refined. Further cultural and organizational adaptation to each country and/or setting is needed to tailor and refine the toolkit.

## Conclusions

Strengthening UH and PHC through transformative education has been at the core of a PAHO/WHO “call for action” for preparing a competent global nursing and midwifery workforce. Building capacity in LAC countries, many with low ratios of nurses and midwives to population and great variation of professional entry-level education, is paramount. Based on a systematic review of available literature, and recognizing that substantial educational differences occur among programs, the EQI toolkit is poised to be adaptable to individual characteristics or circumstances within any nursing or midwifery undergraduate school program. The toolkit offers a dynamic model for a QI plan that could be adopted by educational programs in LAC countries to address weaknesses in UH and PHC within the framework of transformative education and IPCP. Inherent in this work is the acknowledgement that nurses and midwives have important leadership roles to play in health promotion, disease prevention, and reducing morbidity and mortality. A strength of this toolkit stems from its development in partnership with international stakeholders. Future activities will focus on how this educational intervention can be disseminated, evaluated and further improved for broader application.
